# Use of Zeolites in the Capture and Storage of Thermal Energy by Water Desorption—Adsorption Cycles

**DOI:** 10.3390/ma15165574

**Published:** 2022-08-13

**Authors:** Bruno de Gennaro, Angelo Cappi, Maurizio de Gennaro, Nicola Bianco, Alessio Langella, Piergiulio Cappelletti, Antonello Marocco, Paolo Aprea, Michele Pansini

**Affiliations:** 1 ACLabs-Applied Chemistry Labs, Department of Chemical, Materials Engineering and Industrial Production, University of Naples Federico II, P.le V. Tecchio 80, 80125 Naples, Italy; 2 CBC Group SpA, Via Casellina 269, 41058 Vignola, Italy; 3 CeSMA-Laboratory for Insulating Materials Thermal Conductivity Measurements, Centro Servizi Metrologici E Tecnolgici Avanzati Complesso, Universitario San Giovanni, 80146 Naples, Italy; 4 DiSTAR-Dipartimento di Scienze della Terra, Dell’ambiente e delle Risorse, Università di Napoli Federico II, 80126 Naples, Italy; 5 INSTM Research Unit, Department of Civil and Mechanical Engineering, Università degli Studi di Cassino e del Lazio Meridionale, Via G. Di Biasio 43, 03043 Cassino, Italy

**Keywords:** natural zeolites, synthetic zeolites, thermal energy storage, thermal energy waste

## Abstract

In this work, four zeolite-bearing materials (three naturally occurring and one of synthetic origin) were considered for thermal energy capture and storage. Such materials can store thermal energy as heat of desorption of the water present therein, heat that is given back when water vapor is allowed to be re-adsorbed by zeolites. This study was carried out by determining the loss of water after different activation thermal treatments, the water adsorption kinetics and isotherm after an activation step of the zeolites, the intergranular and intragranular porosity, and the thermal conductivity of the zeolite-bearing materials. Moreover, the thermal stability of the framework of the zeolites of the four materials tested was investigated over a large number of thermal cycles. The results indicate that zeolite 13X was the most suitable material for thermal energy storage and suggest its use in the capture and storage of thermal energy that derives from thermal energy waste.

## 1. Introduction

The scientific and technological community considers global heating a matter of major concern due to the possible environmental catastrophes that may stem from it. Such concern is now shared by most of civil society, which appears to be conscious of the possible, tremendous consequences of deliberately ignoring this serious environmental issue. Almost all over the world, political powers have reflected the consciousness of both the scientific and technological community and civil society by setting up international agreements and national acts aimed at limiting global heating [1].

The scientific and technological community has stated that one of the main causes of global heating is the increase in carbon dioxide concentration in the atmosphere, which results in the well-known greenhouse effect. As the main source of carbon dioxide in the atmosphere is human activities, many of the abovementioned international agreements and national acts aim to reduce global heating by limiting carbon dioxide emissions of anthropic origin [2]. This last task appears to be very challenging, as the main source of energy in the world is still the thermal energy obtained from burning fuels of fossil origin [3], which has carbon dioxide as the main product of reaction.

On the basis of these considerations, increasing attention is being paid to renewable energies, in particular solar and discontinuously running energy, and limitation of energy waste. As far as both solar energy and limitation of energy waste are concerned, zeolites may play an outstanding role. Zeolites, in their original, still-valid meaning, are microporous, hydrated, framework aluminosilicates of alkaline or alkaline earth cations [4,5]. The interest in zeolites in the field of the capture and storage of solar energy and limitation of thermal energy waste arises from its peculiarities. Zeolites comprise SiO_4_ or AlO_4_ tetrahedra that link to each other by sharing the O atoms located in the tetrahedron vertices [4,5]. However, the assembly occurs in the space of the SiO_4_ or AlO_4_ tetrahedra takes place leaving a considerable void within the structure of the zeolite itself [4,5]. Such a void forms cavities (called cages) connected by channels, where water molecules and loosely bound cations find their position [4,5]. The amount of water present in such cavities and channels may attain a value of almost 30 wt% [6]. Moreover, the amount of such zeolitic water was found to depend on temperature and water vapor partial pressure as: (i) the lower the temperature and the higher water vapor partial pressure, the higher the amount of water adsorbed in cavities and channels of zeolites; (ii) the higher the temperature and the lower the water vapor partial pressure, the lower the amount of water adsorbed in cavities and channels of zeolites [4,5]. Furthermore, water vapor desorption absorbs the heat necessary to break the physical bindings of water–zeolite, heat that is given back when water vapor is again allowed to be adsorbed by the zeolite, thus recreating the physical bindings of water–zeolite [4,5]. In particular, the physical binding that exists between the zeolite framework and the water molecules arises from the electrical negative charge located on the Al atom present in the zeolite framework and the strongly polar water molecule [4,5].

The energetic features of the water adsorption–desorption process make zeolites proper materials for thermal energy storage. Actually, thermal energy is considered a very poor form of energy on account of: (i) the difficulty of transforming thermal energy into usable work, which results in the drastic energetic penalization of thermal machines; (ii) the practical impossibility of storing thermal energy, which, despite the insulation of the system, results in its dispersion in the external environment over more or less long periods of time.

However, thermal energy from the sun or gaseous streams of combustion products used for other processes (thermal energy waste) could be used to heat a water-saturated zeolite, which would capture the thermal energy necessary to desorb a part of its water vapor. Such an amount of thermal energy could be stored by the zeolite, even for a long time, as the heat of desorption of the water vapor, provided that contact between the partially dehydrated zeolite and the water vapor itself is avoided. The amount of energy of desorption of the water vapor could be given back at the moment when it should be utilized by allowing contact between the partially dehydrated zeolite and water vapor [4,5].

These same considerations have been the focus of several interesting studies [7,8,9,10,11,12,13,14,15,16,17,18,19,20,21,22,23,24,25,26], particularly investigating the storage of solar energy by using zeolites. Despite their valuable contributions, most of these works did not successfully establish practical applications due to their theoretical nature and the insufficiently high activation temperature of zeolites, which could not be attained at the time by using solar energy.

This work is more oriented towards the practical application of zeolites in this field. Its first goal was to re-consider the possibility of using zeolite-bearing materials in solar energy storage, considering the higher activation temperatures of the zeolites that can be attained nowadays due to technological progress [27,28].

Thus, it should be considered that nowadays all over the world, enormous amounts of thermal energy produced by various industrial processes are being dispersed into the environment. For example, the sensible heat of the outlet gaseous stream of a blast furnace is: (i) in small part used to pre-heat the air blown into the blast furnace itself; (ii) in larger part dispersed into the environment, thus giving rise to thermal pollution.

In practice, a considerable part of the sensible heat of a similar gaseous stream, nowadays dispersed in the atmosphere, could be used to activate a water-saturated zeolite. This operation would allow one to store this thermal energy as the heat of water vapor desorption and would allow its use at the moment when it is needed. Thus, the second goal that this work intended to fulfill is evaluating the use of zeolite-bearing materials in the storage of thermal energy coming from thermal energy waste.

On the basis of the previous considerations, four different low-cost, zeolite-bearing materials, both of natural and synthetic origin, were tested in various water vapor desorption–adsorption tests to ascertain their ability to store thermal energy from the sun and from thermal waste. The considerations which guided the choice of these materials are the following: (i) two of these materials, bearing chabazite and phillipsite, are naturally occurring and widespread in Italy; thus, we are trying to promote the natural resources of our country; (ii) the third material bears clinoptilolite, which is the most spread natural zeolite in the world; (iii) the fourth material is a synthetic zeolite (13X), well-known and widely used in industrial processes; it exhibits low cost of production (also thanks to Chinese production, made by companies capable of supplying large quantities of 13X zeolite per month at actual prices between 1430 and 1800 USD/ton with a minimum order of 1–2 tons) and contains a large amount of water, which can be very useful in thermal energy capture and storage (vide infra).

The zeolite-bearing materials of natural origin studied in this work are well-known as they are already used in a number of practical applications such as: environmental protection studies [29,30,31,32,33], ceramization aiming at tile production [34,35,36], production of lightweight aggregates to be used for concrete manufacture [37,38,39], oenological refining processes [40], immobilization of radionuclides [41], additive in animal diet [42], drug carriers [43], and building materials [44].

## 2. Materials and Methods

### 2.1. Materials

The following four zeolite-bearing materials were tested in this work:

(1) PHIL 75: a volcaniclastic material bearing phillipsite (framework type PHI [45]) and chabazite (framework type CHA [38]) from Gallo di Comiziano (outskirts of Naples, Campania region, southern Italy), marketed by Italiana Zeoliti S.R.L.;
(2) CHAB 70: a volcaniclastic material bearing prevailingly chabazite (framework type CHA [45]) from Pian di Rena Sorano (outskirts of Grosseto, Tuscany region, central Italy), marketed by Italiana Zeoliti S.R.L.;
(3) CLINO A: an epiclastic material bearing prevailingly clinoptilolite (framework type HEU [45]) from Hust (Transcarpathian region, western Ukraine), marketed by Italiana Zeoliti S.R.L.;
(4) Synthetic zeolite 13X (framework type FAU [45]), produced in two different grain sizes: 1.5 mm and 3.5 mm by Zibo Yinghe Chemical Co. Ltd. (Zibo, Shandong, China), imported in Italy by Italiana Zeoliti S.R.L.

Qualitative and quantitative phase analysis of materials 1–3, reported in Table 1, was performed by means of X-ray powder diffraction (XRPD and QXRPD, respectively) using a Malvern Panalytical X’Pert Pro diffractometer equipped with a RTMS X’Celerator and = X’Pert High Score Plus 3.0c software (Malvern PANalytical, Almelo, The Netherlands) [46].

The operating conditions were: CuK radiation, 40 kV, 40 mA, a 2 theta range from 4 to 70 theta, an equivalent step size of 0.017 2 theta, and an equivalent counting time of 120 s per step. The data sets were analyzed using the RIR/Rietveld method [47,48] with an internal standard and the TOPAS 5 software (BRUKER AXS Company, Karlsruhe, Germany). Powders with grain size < 10 micron were obtained using a McCrone micronizing mill (agate cylinders and wet grinding time of 15 min; Retsch-Alle, Haan, Germany). XRPD measurements (before and after thermal treatments) were performed on powders previously conditioned for at least 24 h in an R.H. 50% environment. Rietveld quantitative analyses could easily detect any variation in the amorphous content due to the fact that an α-Al_2_O_3_ internal standard (1 micron, Buehler Micropolish) was added to each sample at a rate of 20 wt%.

Starting atomic coordinates for Rietveld quantitative evaluation of identified phases were taken from the literature [49]. The background profile was fitted using a Chebyshev polynomial function with variable number of coefficients (5–12); diffraction peak profiles were modeled refining crystallite size and strain (Lorentzian contribution) coefficients and two Gaussian coefficients. Unit cell parameters along with weight fractions were also refined. Preferred orientation (PO) was treated using the March–Dollase approach [50] whenever needed. All agreement index Rwps were below 8. As evident, PHIL 75 contains similar amounts of phillipsite and chabazite (21 and 27 wt%, respectively), whereas CHAB 70 contains far more chabazite (52 wt%) than phillipsite (4 wt%). CLINO A contains prevailingly clinoptilolite (63 wt%).

Materials 1–3 also contain small amounts of another zeolitic phase: analcime (framework type ANA [45]). The sample of synthetic zeolite 13X used in this work contained about 95 wt% zeolite X, and the rest was prevailingly zeolite A (framework type LTA [45]).

**Table 1:** Mineralogical composition of quarry materials.

	Samples		
Mineral Name	PHIL 75	CHAB 70	CLINO A
Php	21	4	-
Cbz	27	52	-
Cpt	-	-	63
Anl	1	3	-
Fsp	27	14	3
Pl	3	2	1
Aug	5	5	-
Bt	1	2	1
Gls and DCM	15	18	17
Cal	n.d.	n.d.	-
Qz	-	-	15
Total	100	100	100
References	[46]	[46]	[46]

Php = Phillipsite, Cbz = Chabazite, Cpt = Clinoptilolite, Anl = Analcime, Fsp = K-Feldspar, Pl = Plagioclase, Aug = Augite, Bt = Biotite, Gls and DCM = Amorphous fraction and disordered clay minerals, evaluated by difference, Cal = Calcite, Qz = Quartz (Whitney and Evans, 2010 [51]).

The chemical analyses of the zeolites contained in materials 1–4 are reported in Table 2.

Quantitative micro-chemical analyses were carried out by scanning electron microscopy coupled with energy dispersive spectroscopy (SEM and EDS at DiSTAR; Zeiss Merlin VP Compact and JEOL JSM-5310 coupled with Oxford Instruments Microanalysis Unit equipped with an INCA X-act detector; Carl-Zeiss-Strasse, Oberkochen Germany and Jeol Ltd., Tokyo, Japan, respectively). Measurements were performed with an INCA X-stream pulse processor (using a 15 kV primary beam voltage, 50–100 A filament current, variable spot size, from 30,000 to 200,000× magnification, 20 mm WD, and 50 s net acquisition real time). The INCA Energy software (Oxford Analytical Services Ltd, UK) was employed using the XPP matrix correction scheme and the pulse pile-up correction. The quant optimization was carried out using cobalt (FWHM—full width at half maximum peak height—of the strobed zero = 60–65 eV).

The following Smithsonian Institute and MAC (Micro-Analysis Consultants Ltd., Saint Ives, UK) standards were used for calibration: diopside (Ca), fayalite (Fe), San Carlos olivine (Mg), anorthoclase (Na, Al, and Si), rutile (Ti), serandite (Mn), microcline (K), apatite (P), fluorite (F), pyrite (S), and sodium chloride (Cl). Precision and accuracy of EDS analyses were reported in [45].

The chemical composition of the various zeolites of materials 1–3 is similar to that of the precursor (glass) from which they originated. In particular, phillipsite and chabazite come from a glass whose petrographical composition ranges from latite to trachyte [52,53,54,55], and clinoptilolite originates from a more acidic precursor with the composition ranging from dacitic to rhyolitic [56].

Original rocks were crushed, ground, and sieved so as to obtain a fraction with grain size between 1 and 3 mm. These 1–3 mm grain size fractions were used for further experiments and were again subjected to quantitative phase determinations, reported in Table 3.

A moderate reduction in the total zeolite content for PHIL 75 (from 49 to 46 wt%), a larger reduction for CLINO A (from 63 to 53 wt%), and an even larger reduction for CHAB 70 (from 59 to 40 wt%) were recorded. The reduction in total zeolite content is related to the fact that the production of the grain size fraction 1–3 mm gave rise to the formation of a finer grain size fraction richer in zeolites which was discarded, thus reducing the whole zeolite content of the remaining material. Zeolite 13X was supplied in a grain size fraction of 1.8–2.4 mm.

The materials investigated in this work, prior to whatever experiment was performed, were kept at least 3 days in an environment with about 50% R. H. (created by a saturated Ca(NO_3_)_2_ aqueous solution [57,58]) to allow water saturation of zeolites.

### 2.2. Modalities of Determination of Weight Loss

The weight loss of materials 1–4 after thermal treatment at various activation temperatures (100, 150, 200, 250, and 300 °C), prolonged for different times (1, 2, 3, 5, and 10 h), was determined by subjecting materials 1–4 themselves to thermogravimetric (TG) analysis (10 °C/min heating rate) by using a NETZSCH thermal analyzer (NETZSCH Holding, Selb, Germany), model STA 449 F3 Jupiter [57].

The ability of materials 1–4 to adsorb and desorb water vapor in repeated heating–cooling cycles was investigated as follows. The weight loss of materials 1–4, after bringing them from room temperature to 250 °C (10 °C/min heating rate) and keeping them at this temperature for 2 h, was determined by TG analysis. Then, materials 1–4 were allowed to fully rehydrate as previously described (**vide supra**), brought again to 250 °C with the same previous modalities, and kept again for 2 h at this temperature to determine the final weight loss in this second cycle. Moreover, samples of materials 1–4 were subjected to the following thermal treatments: (i) heated at 50 °C (10 °C/min heating rate) and kept at this temperature for 10 h; (ii) heated at 250 °C (10 °C/min heating rate) and kept at this temperature for 2 h; (iii) heated at 350 °C (10 °C/min heating rate) and kept at this temperature for 1 h. These samples were allowed to fully rehydrate, and then subjected to XRPD analysis with quantitative determination of the various phases, as mentioned above. Finally, a sample of zeolite 13X was: (i) heated at 250 °C (10 °C/min heating rate); (ii) kept at this temperature for 2 h; (iii) cooled down to room temperature; (iv) allowed to rehydrate as described above; (v) again heated at 250 °C (10 °C/min heating rate). This thermal cycle was iterated 60 times. The sample of zeolite 13X was subjected to XRPD analysis after 30 and 60 cycles, and its water loss was determined.

### 2.3. Water Adsorption Kinetics

The water adsorption kinetics of the various materials after they were subjected to an activation thermal treatment were determined as follows. Weighed samples of materials 1–4 were placed on a pan and suspended under a gold-coated wolfram spring (resolution 0.120 mg) in a glass vessel of a McBain thermobalance. The vessel was evacuated to a pressure of *p* < 1 × 10^−3^ Pa, heated up to the activation temperature of 250 °C (10 °C/min heating rate), and kept at this temperature for 2 h. Then, the temperature of the system was brought back to 25 °C, and water vapor was allowed to enter the vessel at a pressure of 1.6 kPa. Gas pressure was directly measured with a capacitive pressure transducer (Edwards Datametrics 1500), while the amount of adsorbed water was calculated by measuring the spring elongation by means of a cathetometer with a sensitivity of 0.05 mm. The temperature of the adsorption chamber was controlled by a Heto Thermosetting unit. The thermal activation of the sample was performed **in situ** prior to the measurement by using an Edwards turbomolecular pump and a toroidal oven.

The kinetics of water vapor adsorption was determined by recording the amount of water vapor adsorbed by materials 1–4 at various times. These experiments were iterated at 25, 45, and 65 °C adsorption temperature.

### 2.4. Water Adsorption Isotherms

The water adsorption capacity of the various materials after they were subjected to an activation thermal treatment was measured by determining water vapor adsorption isotherms as follows. Weighed samples of CHAB 70 and Zeolite 13X were located on a pan and suspended under a gold wolfram spring in the glass vessel of the same McBain thermobalance, described above. The vessel was evacuated to a pressure of *p* < 1 × 10^−3^ Pa, heated up to the activation temperature of 150 °C (10 °C/min heating rate), and kept at this temperature for 2 h. Then, the temperature of the system was brought back to 25 ° C (isotherm temperature), and water vapor was allowed to enter the vessel at increasing pressures (0.067, 0.27, 0.53, 0,80, 1.07, 1.33, and 1.60 kPa) once 30 min had elapsed after each water vapor addition. The amount of adsorbed water vapor was evaluated through the elongation of the gold wolfram spring.

This same procedure was repeated for all four materials by changing the following operative conditions: (i) the activation temperature was 250 °C; (ii) the adsorption isotherm temperatures were 25, 45, and 65 °C. Adsorption isotherms were adapted to Langmuir’s model, which allows the interpretation of a predominantly monolayer adsorption process:

(1) $$q_{e}=q_{e}^{max}\frac{bp}{1+bp}$$

where $q_{e}^{max}$ (mmol g^−1^) is the maximum adsorption capacity at equilibrium and *b* is the affinity constant.

### 2.5. Porosity Determinations

Zeolite 13X was subjected to porosity determinations. In particular, the porosity existing inside the zeolite grains (intragranular porosity) was determined. Moreover, grains of zeolite 13X were accommodated into a vessel so as to mimic the loading of this material in a device to be used in thermal energy storage, and the void existing between the various grains (intergranular porosity) was determined. The procedure for the determination of intergranular porosity (P_inter_) was carried out in ref. [59], according to the CNR B.U. 22, ASTM D1188, and BS 1377 T15/E standards, and it can be summarized as follows: the material was placed in a cylindrical container of known volume (*Vt*) and weight equipped at the ends with perforated caps, ensuring its compaction by vibration on an electromechanical device (belonging to a vibrating sieve device). The system was then saturated by immersion in demineralized water for 72 h. After careful external drying of the granules for the elimination of intergranular capillary water, the sample was repositioned in the cylindrical containers by compaction on the same electromechanical device. Subsequently, the weight of the saturated granules and cylindrical containers and the total volume of the grains (*Vs*) were measured by hydrostatic weighing. In all experiments, the water temperature was maintained at 24.5 °C, with a density of 0.993 kg/L. Finally, the calculation of the intergranular porosity by means of the elementary relation was done using the following equation:

(2) $$P_{\mathrm{inter}}=\frac{Vt-Vs}{Vt}\;$$

The procedure for determining the total porosity (Pt) and subsequently the intragranular porosity (P_intra_) was carried out in ref. [59], according to the CNR—UNI 100008, ASTM D 2216, CNR B.U. 64, and ASTM D 854 standards and is as follows: the test material was dried and the dry weight and weight of the dry volume unit (γ_dry_) determined. After the sample was reduced to a powder by grinding, appropriate pycnometer weighing operations were followed to determine the specific weight of the solid particles (Gs), measured in g/cm^3^. The calculation of the total porosity (Pt) was finally performed by means of the relation:

(3) $$\mathrm{Pt}=1-(\frac{\gamma_{dry}}{Gs})$$

From the values obtained with the previous relationship, we proceeded to calculate the intragranular porosity (Pintra):

P_intra_ = Pt − P_inter_ (4)

### 2.6. Thermal Conductivity Determinations

Thermal conductivity measurements were performed at the Laboratory for Insulating Materials Thermal Conductivity of CeSMA Measurements on 13X zeolite in the form of spherical grains (diameter 1.8 ÷ 2.4 mm) at various temperatures (40, 80, 120, and 160 °C) using a NETZSCH Guarded Hot Plate (GHP) 456 Titan System, high-temperature version, according to the following procedure. A plate with area A, which became hot due to electrical power supply, was sandwiched between two layers of zeolite 13X with the same thickness t. The two layers necessary to carry out each measurement were prepared by inserting the spherical grains of zeolite in two square TEFLON frames that were 300 mm × 300 mm × 40 mm, with a free internal area of 220 mm × 220 mm. A guard ring was located all around the hot plate to minimize the lateral heat dispersion. Two other plates, placed above and below the samples to hold the layers of zeolite spheres in place, were kept at a low temperature by liquid nitrogen. All plate temperatures were controlled through the heating (power supply) and cooling (liquid nitrogen) systems so that a defined temperature difference ΔT—set by the user—was established between the hot and cold plates, i.e., through the thickness of the sample. This ΔT gave rise to the thermal flux Q through the layers of zeolite 13X. Such thermal flux Q was the thermal power input to the hot plate, generated electrically thanks to the Joule effects. Once the steady-state conditions was attained, the thermal conductivity λ of zeolite 13X was given by the Fourier law:

λ = (Q·t)/(ΔT·2A)(5)

A is the front area of the sample, generally equal to the hot plate area; the factor 2 is present because the measure was performed on two samples.

## 3. Results

### 3.1. Weight Loss

Table 4 reports the weight loss of materials 1–4 after thermal treatment at various activation temperatures (100, 150, 200, 250, and 300 °C) prolonged for different times (1, 2, 3, 5, and 10 h). The most striking features of these data are the following:

(1) Weight loss of materials 1–4 increased according to the sequence CLINO A < PHIL 75 < CHAB 70 << zeolite 13X, ceteris paribus. It must be noted that the weight loss of zeolite 13X was often higher than double that of CHAB 70, which in turn was decidedly higher than that of PHIL 75 and CLINO A, ceteris paribus.
(2) Weight loss of materials 1–4 increased with increasing activation temperature and time, as one may expect. However, the dependence from the time did not appear so strong, as weight loss usually increased by 10–20% (with some exceptions) when increasing the time of thermal treatment from 1 to 10 h. Unlike this last parameter, the activation temperature seemed to play a crucial role in affecting the weight loss, as it increased to a far larger extent (zeolite 13X and CHAB 70 about 200%, PHIL 75 about 250%, and CLINO A about 500%, from 100 to 300 °C).
(3) The extent to which the weight loss of materials 1–4 increased with the activation temperature and time strongly differed between materials.
(4) Comparing the weight loss data in Table 4 with the water content of zeolites in Table 2 suggests that most of the zeolitic water was lost at 300 °C. It must be borne in mind that the water content of zeolites, reported in Table 2, refers to pure zeolites and thus, such values must be re-scaled to the total zeolite content of the various zeolite-bearing materials (PHIL 75 = 46%, CLINO A = 53%, CHAB 70 = 40%, and zeolite 13X = almost 100%).

Table 5 concerns the ability of materials 1–4 to adsorb and desorb water vapor in repeated heating–cooling cycles. Its first line reports the weight loss of materials 1–4 after bringing them from room temperature to 250 °C (10 °C/min heating rate) and keeping them at this temperature for 2 h. The second line reports the weight loss of the same samples of materials 1–4, used in the first cycle, after they were allowed to fully rehydrate (see experimental section), brought again to 250 °C with the same previous modalities, and kept again for 2 h at this temperature. Differences lower than 6% were recorded for the same zeolite-bearing material in the two different cycles.

### 3.2. Thermal Stability of Zeolites

Water loss by heating may affect the stability of the microporous zeolite framework [4,5,60]. This issue was investigated by subjecting to XRPD and by performing the quantitative phase determination of materials 1–4 after the various thermal treatments described in the experimental section. These data are summarized in Table 6. It is evident that all the zeolitic phases present in materials 1–4 were scarcely damaged by the various thermal treatments to which they were subjected, with the exception being phillipsite. The amount of this zeolite was roughly halved by such thermal treatments.

No damage was found for zeolite 13X after the same treatments.

Finally, Table 7 compares the water loss of a sample of zeolite 13X after heating at 250 °C and being kept at this temperature for 2 h, with the water loss recorded after 30 and 60 thermal cycles (see experimental section). Differences in water loss were meaningless and are included within the experimental error. The integrity of zeolite structures of sample 13X was confirmed by the XRPD analysis.

### 3.3. Kinetics of Adsorption

**Figure 1:**
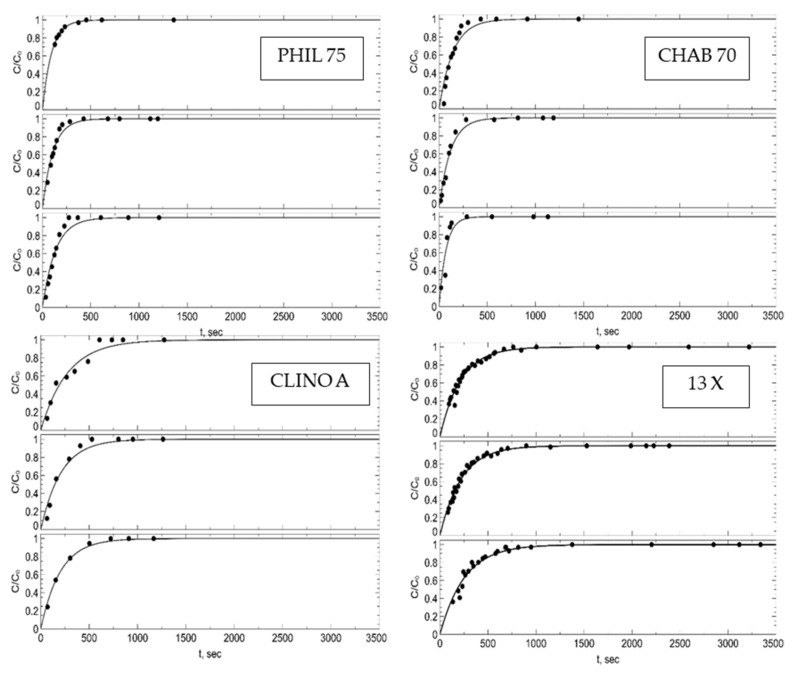
Kinetic curves of adsorption of water vapor. For each sample or material from top to bottom: 25 °C; 45 °C; 65 °C.

Kinetic curves of water adsorption are reported in Figure 1. The trend of these curves appeared similar in all of them. However, the values of the time sufficient to attain 80% of the water adsorption equilibrium value and 100% of the water adsorption equilibrium value decidedly differed from each other. These values were: (CHAB 70) 170 and 220 min, (PHIL 75) 220 and 300 min, (CLINO A) 300 and 540 min, and (zeolite 13X) 250 and 750 min, respectively. It can be noticed that the times necessary to attain such water adsorption equilibrium values increased according to the following order: CHAB 70 < PHIL 75 < CLINO < zeolite 13X.

### 3.4. Adsorption Isotherms

**Figure 2:**
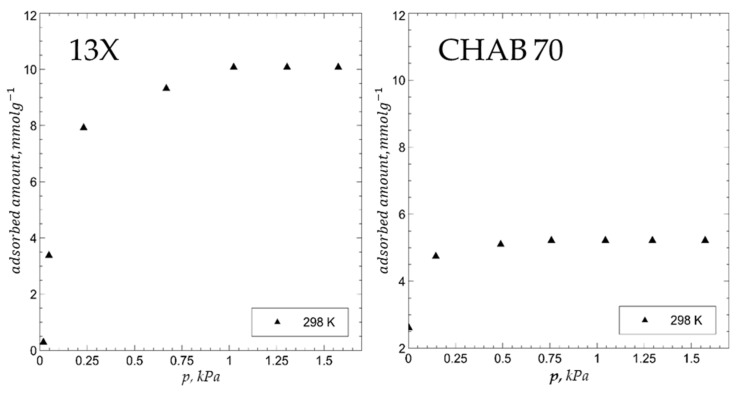
Water adsorption isotherm at 25 °C on zeolite 13X and CHAB 70 after activation at 150 °C.

**Figure 3:**
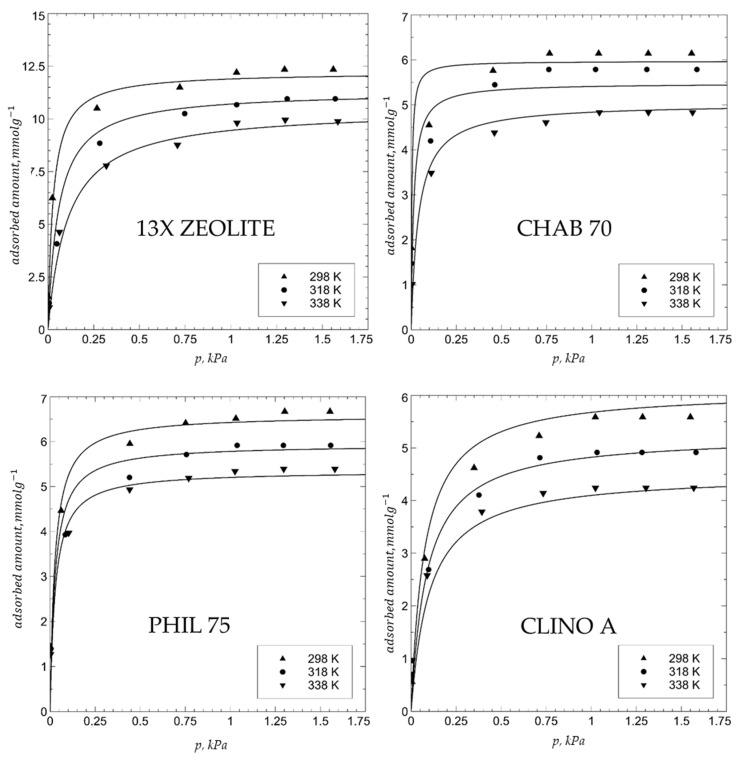
Water adsorption isotherm at 25, 45, and 65 °C on zeolite 13X, CHAB 70, PHIL 75, and CLINO A after activation at 250°C. Points = experimental; lines = Langmuir model.

The water adsorption isotherm at 25 °C on zeolite 13X and CHAB 70 after activation at 150 °C (see experimental section) is reported in Figure 2, whereas the water adsorption isotherm at 25, 45, and 65 °C on materials 1–4 after activation at 250 °C (see experimental section) is reported in Figure 3.

The most striking features of the adsorption isotherms are the following:

(1) The adsorption equilibrium value was attained at a water vapor pressure of about 1 kPa, and about 80% of this value was attained at a water vapor pressure of about 0.3 kPa.
(2) The adsorption equilibrium value of materials 1–4 was consistent with the recorded weight loss data (see above), which means that the amount of water lost in the activation step was acquired in the subsequent adsorption step.
(3) Water adsorption values increased according to the following sequence: CLINO A (5.5 mmol g^−1^ at 25 °C) < CHAB 70 (6.0 mmol g^−1^ at 25 °C) ≈ PHIL 75 (6.2 mmol g^−1^ at 25 °C) << 13X (12.0 mmol g^−1^ at 25 °C), ceteris paribus; in particular, water adsorption values of zeolite 13X were almost double those of CHAB 70 and PHIL 75.
(4) Water adsorption values decreased with temperature; in particular, a reduction of about 20% was recorded from 25 to 60 °C.
(5) 13X zeolite reached the maximum adsorption values of natural zeolites (about 6 mmol g^−1^ at 25 °C) already at 0.1 kPa of pressure in the test vessel.
(6) The Langmuir model satisfactorily fit the various adsorption isotherms.

### 3.5. Intergranular and Intragranular Porosity and Thermal Conductivity of Zeolite 13X

Table 8 reports the intergranular porosity (see experimental section), the intragranular porosity (see experimental section), and the total porosity of the system (total void present in the system, sum of the intergranular and intragranular porosity) of zeolite 13X.

**Figure 4:**
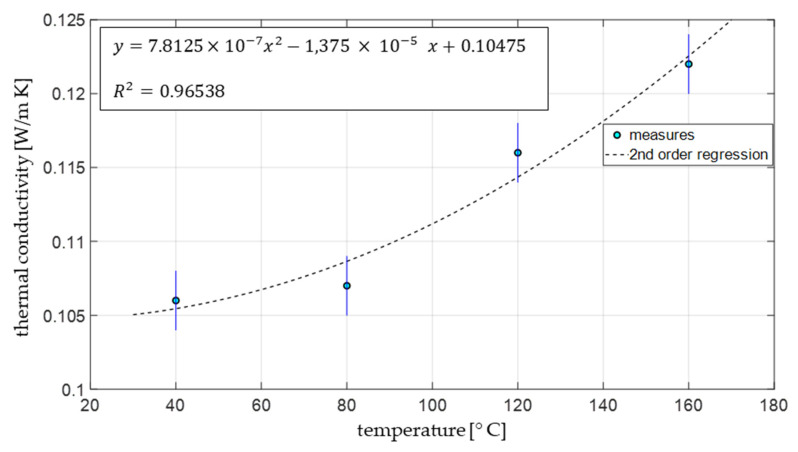
Measurement results: thermal conductivity (λ).

The values of the first two types of porosity were around 30%, which means that the total void in the device where zeolite 13X found accommodation was higher than 60%. These voids affected the values of the thermal conductivity λ, measured at various temperatures, which are reported in Figure 4.

Thermal conductivity **λ** was found to increase with temperature with a parabolic trend (by about 15% from 40 to 160 °C).

## 4. Discussion

A careful inspection of compositional data of materials 1–4 allowed us to infer the following. Naturally occurring, zeolite-bearing materials 1–3 exhibited a zeolite content of about 50–60%. Such contents were essentially further reduced by the grinding and granulation process owing to the formation of fine powders richer in zeolites. The sample of industrial, synthetic zeolite 13X, used as material 4 in this work, contained about 95% zeolite 13X and the rest was prevailingly zeolite A. Thus, the zeolite content of material 4 was slightly lower than 100%. Moreover, the water content of various zeolites of materials 1–4 increased according to the following sequence: PHIL 75 < CLINO < CHAB 70 < zeolite 13X. This sequence may be explained by the following considerations. The water content of materials 1–4 is related to the % of zeolite present in the material and to the void of the zeolites present therein. As far as the void is concerned, the higher this parameter, the lower the framework density of the zeolite (FAU: 12.7, CHA 14.6, PHI 15.8, and HEU 17.0 atoms with tetrahedral coordination (Si or Al) per A^3^ [45]), which means that the void present in the zeolites increased according to the sequence HEU < PHI < CHA < FAU. Thus, the combination of the zeolite content of the material and its void resulted in the previously reported sequence of water content (PHIL 75 < CLINO < CHAB 70 < zeolite 13X).

Both sets of data, concerning the zeolite amount and zeolite water content, concurred in that zeolite 13X exhibited a decidedly higher water content than materials 1–3. This result is highly significant, as the ability of a material to capture and store thermal energy on the basis of a reversible water desorption–adsorption process is proportional to the water content of the material itself, at least according to a first approximation.

Data concerning weight loss of materials 1-4 absolutely confirmed the previous considerations: zeolite 13X exhibited a weight loss (in practice, water loss) far higher than materials 1–3 no matter the activation temperature and the time of activation thermal treatment. With regards to these last two parameters, activation temperature seemed to play a far more crucial role than the duration time of the thermal treatment in resulting in higher water losses (see Table 4). Thus, this last issue seems to suggest activation temperatures as high as possible (250–300 °C) and short duration times of such thermal treatments (1–2 h).

Unlike the data concerning the weight loss of materials 1–4, which appeared more favorable for zeolite 13X than for materials 1–3, data concerning the ability of materials 1–4 to adsorb and desorb water vapor in repeated heating–cooling cycles showed a small reduction (lower than 6%) in such ability to a similar extent for materials 1–4.

Interesting results regarding the thermal stability of zeolites involved in this study were achieved. Repeated thermal cycles with the related water loss did not noticeably affect the stability of the framework of chabazite, clinoptilolite, and zeolite 13X unlike that of phillipsite, whose content was roughly halved by the thermal treatments. Despite this, phillipsite, although damaged by dehydration on heating, seemed to retain its ability to adsorb and desorb water to about the same extent it exhibited prior to the occurrence of the structural damage originated by the thermal treatment. This issue might be explained as follows. Dehydration on heating affected the stability of the framework of phillipsite, whose diffraction peaks in the XRPD pattern result were largely reduced in intensity. However, thermal motions and dehydration affected only the degree of order of the phillipsite structure but not its microporosity, which remained substantially unaltered. Thus, the partially amorphous phillipsite, deriving from the thermal treatment and subsequent dehydration, almost totally retained its ability to adsorb–desorb water vapor. Further investigations of this point would be very interesting but would go beyond the aims of this work and thus will be the subject of forthcoming work. As far as the ability to capture and store thermal energy in repeated heating–cooling cycles by the various zeolite-bearing materials studied in this work, it can be said that it remained almost unaltered with the increasing number of thermal cycles for all materials 1–4. However, the thermal stability of the sample zeolite 13X must be highlighted in particular, as it was tested after 60 heating–cooling cycles, and it was found practically unaltered.

Kinetic data in Figure 1 suggest that times of from 170 to 250 min and from 260 to 750 min were necessary to attain 80% of the water adsorption equilibrium value and 100% of the water adsorption equilibrium value, respectively. These times increased according to the sequence CHAB 70 < PHIL 75 < CLINO < zeolite 13X. This sequence is probably a result of the combined effect of pore size and water amount, since larger pores should ease equilibration but a higher water capacity should take longer to equilibrate.

Even water adsorption isotherms supplied interesting information concerning the ability of materials 1–4 to capture and store thermal energy by a water vapor desorption–adsorption process. Firstly, the maximum water vapor adsorption capacity was attained at quite a low water vapor partial pressure such as 1 kPa. This means that samples of materials 1–4, activated by a short (even 1 h) thermal treatment at 250–300 °C, could be fully and rapidly rehydrated by flowing through the activated zeolite-bearing material humid air.

The fact that water vapor adsorbed by activated zeolite 13X was about double that adsorbed by the other zeolite-bearing materials perfectly agrees with the data of zeolite content and water content of zeolites previously discussed and strongly suggests zeolite 13X as the most suitable material for thermal energy storage. Actually, the only points that are in favor of materials 1–3 are (i) the reduced time needed to attain adsorption equilibrium, and (ii) the fact that naturally occurring zeolite-bearing materials exhibit a lower cost than synthetic zeolites. However, the extent to which these two points are in favor of materials 1–3 is not so dramatic as to completely upset all the results so far presented. Thus, the remaining experiments were performed only on zeolite 13X, as the collected results were sufficient to establish that this material was the most suitable for thermal energy storage.

The values of the thermal conductivity λ of zeolite 13X measured at various temperatures are consistent with the data of ref. [61] and similar to those of other porous ceramic materials [62,63,64]. Such values were found to increase by increasing the temperature, ranging between 0.106 and 0.122 Wm/K at 40 and 160 °C, respectively. To further increase the value of the thermal conductivity λ, action can be taken. Firstly, a wider grain size distribution of the particles of zeolite 13X would ensure a better occupancy of the space where the zeolite grains are located, as smaller grains may establish a position in the space left available by the larger grains. Operating in such a way, the intergranular porosity of zeolite 13X would be reduced and thus, the thermal conductivity λ would be enhanced. Moreover, manufacturing PTFE–zeolite composites similar to those described in ref. [13] may result in a reduction in the whole thermal resistance of the devices in which the zeolite-bearing materials are located.

A last point still needs to be discussed. Ref. [6] showed indubitably that the water content of a zeolite depends on its cation population. In particular, it was found that the water content of a zeolite was higher the smaller the cations were and the higher the valence of the cations present in the zeolite framework was (to counterbalance the negative charges arising in the anionic framework from the substitution of Si with Al). Thus, one might think about exchanging zeolite 13X with small cations with a valence higher than one to increase the water content of the zeolite. In a previous work, the authors carried out an evaluation of the cation content of some zeolites in terms of their water adsorption behavior, finding such a correlation between the cationic species and the isosteric heat of adsorption [65], although besides this evidence, the opportunity of a cation exchange step should not be taken for granted. The zeolite would exhibit a larger water content, but this water would be more strongly retained by the cations of smaller dimensions and higher valence owing to the higher electrical density charge. It appears possible that for high activation temperatures of the zeolite, higher water content of the zeolites, arising from a cation population of smaller radius and higher valence, although more strongly retained, would give better results in thermal energy storage. However, for low activation temperatures of the zeolite, a lower water content, more weakly retained and deriving from a cation population of higher radius and lower valence, would be more beneficial in thermal energy storage.

## 5. Conclusions

This investigation drew on a number of interesting considerations, which may be of great help in setting up devices based on the desorption–adsorption of water vapor on zeolite-bearing materials for thermal energy capture and storage.

Firstly, zeolite 13X appeared the most suitable zeolitic material for thermal energy capture and storage owing to: (i) the higher zeolite content; (ii) the higher water content; (iii) the cost, which, although higher than that of natural zeolites, remains sufficiently low. Moreover, zeolite 13X appeared more suitable than the naturally occurring materials 1–3 studied in this work and is probably more suitable than any synthetic zeolite-bearing materials. Although some other synthetic zeolites exhibiting higher water content were found, they very likely would cost far more than zeolite 13X, thus rendering their use less convenient.

Secondly, the strong dependence of water loss on the activation temperature, and in particular, the fact that an activation temperature of about 300 °C allowed the dehydration of zeolite to a large extent (80–90%), suggest that 300 °C is an optimal activation temperature of zeolites to be used in thermal energy storage based on water vapor desorption–adsorption processes. This temperature is attained with no problem in the outlet gaseous stream deriving from fuel combustion reactions needed in many industrial processes, but it cannot be attained yet by concentrating the energy of sunlight. Thus, the use of zeolite 13X is more suitable to capture and store the thermal energy contained in the thermal energy waste described in Section 1. As far as solar energy storage is concerned, the highest temperature that can be attained by concentrating such energy through current technologies is about 150 °C. Thus, given the strong dependence of the water desorbed from the zeolites on their activation temperature, an activation temperature of about 150 °C would likely result in the capture of an insufficiently large amount of solar energy, thus rendering the whole process uneconomical. In conclusion, to use zeolite-bearing materials for solar energy storage, we have to hope that the technological processes of concentration of solar energy will, in a few years, allow us to attain temperatures higher than 150 °C.

Another interesting consideration concerns the shape of the devices where the zeolite-bearing material to be used for thermal energy storage should be located. The suboptimal thermal conductivity λ suggests locating the zeolite-bearing materials in vessels with one dimension largely prevailing over the other two dimensions in order to maximize their specific surface area, which would largely enhance thermal exchange.

## Figures and Tables

**Table 2:** Chemical composition of zeolites present in the selected materials.

	PHIL 75	CHAB 70	CLINO A	13X ZEOLITE
SiO_2_	54.10	49.85	65.30	41.36
TiO_2_	0.19	0.12	0.15	0.12
Al_2_O_3_	18.11	18.21	12.15	26.05
Fe_2_O_3_	0.39	0.27	0.18	0.18
MnO	0.06	0.12	0.07	0.13
MgO	0.11	0.32	1.03	0.49
CaO	4.50	5.60	3.80	1.82
Na_2_O	0.76	0.56	0.28	12.38
K_2_O	8.64	6.19	2.11	0.57
H_2_O	13.15	18.75	14.81	17.11
Total	100.01	99.99	99.88	100.21

**Table 3:** Mineralogical composition of the granulates.

	Samples		
Contained Phases	PHIL 75	CHAB 70	CLINO A
Php	20	3	-
Cbz	25	35	-
Cpt	-	-	53
Anl	1	2	-
Fsp	24	30	15
Pl	4	2	1
Aug	4	6	-
Bt	2	2	2
Gls and DCM	19	20	16
Cal	1	n.d.	3
Qz	-	-	10
Total	100	100	100
References	[46]	[46]	[46]

Php = Phillipsite, Cbz = Chabazite, Cpt = Clinoptilolite, Anl = Analcime, Fsp = K-Feldspar, Pl = Plagioclase, Aug = Augite, Bt = Biotite, Gls and DCM = Amorphous fraction and disordered clay minerals, evaluated by difference, Cal = Calcite, Qz = Quartz (Whitney and Evans, 2010 [51]).

**Table 4:** Weight loss of materials 1–4 after thermal treatment at various activation temperatures prolonged for different times.

**Dwell (h)**	**PHIL 75 (% W** **eight Loss)**				
	100 °C	150 °C	200 °C	250 °C	300 °C
1	2.25	4.93	6.51	7.38	8.00
2	2.33	5.05	7.77	7.74	8.04
3	2.42	5.37	8.03	8.07	8.26
5	2.46	5.55	8.54	8.43	8.73
10	2.67	5.76	8.86	8.61	9.29
**Dwell (h)**	**CHAB 70 (% W** **eight Loss)**				
	100 °C	150 °C	200 °C	250 °C	300 °C
1	3.25	5.72	7.63	8.00	8.50
2	3.26	5.87	8.05	8.96	9.22
3	3.28	6.17	8.09	8.97	9.52
5	3.30	6.22	8.29	8.99	9.72
10	3.33	6.35	8.75	9.05	9.98
**Dwell (h)**	**CLINO A (% W** **eight Loss)**				
	100 °C	150 °C	200 °C	250 °C	300 °C
1	1.08	5.08	5.87	6.83	7.44
2	1.14	5.12	6.33	7.32	7.78
3	1.27	5.53	6.45	7.38	7.85
5	1.48	5.80	6.68	7.53	7.87
10	1.50	6.53	7.11	7.67	7.83
**Dwell (h)**	**13X Zeolite 1.5 mm (% W** **eight Loss)**				
	100 °C	150 °C	200 °C	250 °C	300 °C
1	4.55	15.07	15.75	16.07	17.30
2	4.99	15.08	15.78	16.07	17.20
3	5.49	15.14	15.87	16.45	17.31
5	6.67	15.17	16.03	16.73	17.45
10	10.83	15.23	16.40	17.73	17.82
**Dwell (h)**	**13X Zeolite 3.5 mm (% W** **eight Loss)**				
	100 °C	150 °C	200 °C	250 °C	300 °C
1	4.17	15.10	16.08	16.64	17.43
2	4.95	15.14	16.19	16.68	17.49
3	5.36	15.19	16.34	16.75	17.55
5	7.00	15.25	16.49	16.85	17.75
10	11.67	15.30	16.82	17.05	18.18

**Table 5:** Comparison between the weight loss values of the samples before and after treatment.

SAMPLES			
PHIL 75	CHAB 70	CLINO A	13X
Weight loss (%) of intact material			
8.52	8.09	9.18	18.47
Weight loss (%) after 250 ° C × 2 h and subsequent rehydration			
9.11	8.14	9.29	18.68

**Table 6:** Evaluation of the damage suffered by zeolites with heat treatment (weight % variation).

	**CHAB 70**			
Phases	As it is	50_10 h	250_2 h	350_1 h
Chabazite	35	31	30	34
Phillipsite	3	2	2	2
Analcime	2	1	1	3
K Feldspar	30	26	24	29
Albite	2	4	2	4
Biotite	2	3	2	3
Calcite	0	0	0	0
Augite	6	5	6	7
Amorphous	20	28	32	18
Total	100	100	100	100
	**PHIL 75**			
Phases	As it is	50_10 h	250_2 h	350_1 h
Chabazite	25	22	22	24
Phillipsite	20	12	12	9
Analcime	1	1	1	1
K Feldspar	24	22	21	17
Albite	4	3	4	5
Biotite	2	2	3	4
Calcite	1	5	2	2
Augite	4	5	5	5
Amorphous	19	28	30	33
Total	100	100	100	100
	**CLINO A**			
Pashes	As it is	50_10 h	250_2 h	350_1 h
Clinoptilolite	53	53	51	42
Quartz	10	12	9	11
Calcite	3	3	3	3
K Feldspar	15	15	14	16
Albite	1	1	1	1
Biotite	2	2	3	2
Amorphous	16	13	19	25
Total	100	99	100	100

**Table 7:** 13X zeolite 1.5 mm: comparison between material mass losses before and after 30 and 60 cycles 40 °C→250 °C × 2 h→40 °C.

13X Zeolite	13X Zeolite after 30 Cycles	13X Zeolite after 60 Cycles
16.66%	16.79%	16.46%

**Table 8:** Porosity of the two grain sizes of 13X zeolite.

Sample Grain Size	1.8–2.4 mm
Intergranular porosity (%)	34.3%
Intragranular porosity (%)	29.5%
Total porosity (%)	63.8%

## Data Availability

All data derived from this research are presented in the enclosed figures and tables. Anyway, data presented are available on the request from the corresponding author. Data and chemical analysis reported as: “Italiana Zeolite (CBC Group Company) Record”, refer to reports of analysis made by Research Laboratories of Italian Universities are available on request from the co-author Prof. Geol. Maurizio de Gennaro responsible for research activities of raw materials of Soc. CBC Group. Data are not publicly available due to commercial confidentiality.
